# Age-related cytokine imbalance in the thymus in sudden infant death syndrome (SIDS)

**DOI:** 10.1038/s41390-023-02809-6

**Published:** 2023-09-07

**Authors:** Dong Qu, Vanessa Preuss, Lars Hagemeier, Lena Radomsky, Kerstin Beushausen, Jana Keil, Schaumann Nora, Benedikt Vennemann, Christine S. Falk, Michael Klintschar

**Affiliations:** 1 Institute of Legal Medicine, Hannover Medical School, Hannover, Germany; 2 Institute of Transplant Immunology, Hannover Medical School, Hannover, Germany; 3 German Center for Infection Research, DZIF, TTU-IICH, Hannover-Braunschweig site, Hannover, Germany; 4 Institute of Pathology, Hannover Medical School, Hannover, Germany

## Abstract

**Background:**

Sudden infant death syndrome (SIDS) has been considered to be triggered by a combination of underlying immune dysregulation and infections. The thymus is a crucial lymphatic organ responsible for T cell development in infancy. We hypothesized that an altered thymic immune status may be detectable by intrathymic cytokine profiling in SIDS.

**Methods:**

27 cytokines in protein lysates of thymus tissue and thymus weights were assessed in 26 SIDS cases and 16 infants who died of other reasons.

**Results:**

Seventeen out of 27 cytokines were increased in thymic tissue of SIDS compared to controls without infections, and the most significant discrepancy was in infants younger than 20 weeks. The thymic cytokine profiles in SIDS cases were similar to those in controls with severe infection; however, the magnitude of the cytokine concentration elevation in SIDS was less pronounced, indicating sub-clinical infections in SIDS. In contrast to SIDS, intrathymic cytokine concentrations and thymus weight were increased with age in control children.

**Conclusions:**

Elevated thymic cytokine expression and thymus weight, as well as impaired age-related alterations in SIDS, may be influenced by subclinical infection, which may play a role in initiating SIDS in infants with a compromised immune response.

**Impact Statement:**

- Increased thymic weight and cytokine concentration may suggest possible subclinical infection in SIDS.
- Elevated thymic weight and cytokine concentration mainly in SIDS cases aged <20 weeks.
- Age-related impairment in the thymic weight and cytokine expression may be impaired by subclinical infection in SIDS.

## Introduction

Sudden infant death syndrome (SIDS)^1^ is the leading cause of death among infants aged 1 month to 1 year in developed countries. Triple risk models suggest that a combination of critical developmental stages, vulnerable infants, and exogenous factors are associated with SIDS.^2,3^ Nevertheless, the exact etiologic mechanism remains unclear. It has been discussed for a long time that an impaired immune response and silent infections may precipitate SIDS.^4–12^ Some studies implied the existence of hyperinflammation, whilst others suggested immunodeficiency.^7,13–17^ Therefore, more information is needed to figure out the precise mechanism that may contribute to an impaired immune response in SIDS.

To this goal, the thymus, which is charged with the development of the immune system during infancy,^18^ comes into view. It is well acknowledged that severe primary immunodeficiency originating from the thymus, such as DiGeorge syndrome,^19^ can result in a weakened response to common infections, ultimately leading to infant mortality.^20^ Regarding sudden deaths of infants, an involvement of the thymus was suspected long before the term SIDS was introduced: According to Dally,^21^ the Austrian forensic pathologist reported an increased weight of the thymus in suddenly deceased infants and coined the term “status lymphaticus”.^22^ Numerous studies extensively investigated thymic weight, histology, etc. despite divergent outcomes.^23–33^ Goldwater et al. discovered an increased thymus weight in SIDS cases, which may be the consequence of subclinical infections.^27^ Several other studies regarding thymus weight observed comparable or contradictory findings.^25,26,31,32^ An article by Varga et al. indicated that immunohistochemistry (IHC) revealed the presence of thymic involution in SIDS, which may be driven by stress.^33^ However, Bajanowski et al. reported an absence of deficits of T or B cells in the thymus and other selected lymphatic organs.^23^

The cytokine network in the thymus is crucial for responding to infection and regulating thymus maturation.^34–36^ Changes in the cytokine network are expected if the thymus displays functional disorders. However, there is a paucity of research addressing changes in the cytokine network between SIDS and control cases. Since probable alterations in thymic weight and histology have been described earlier in SIDS, we hypothesized that these alterations would be accompanied by changes in cytokine and chemokine profiling. This study employed a multiplex cytokine measurement approach to characterize the thymic cytokine profile, which may aid in reflecting the thymus status and clarifying probable SIDS etiology mechanisms.

## Materials and methods

### Study participants

The subjects investigated were 42 control and SIDS cases autopsied at the Institute of Legal Medicine, MHH, between 2010 and 2020. Approval was given by the local ethics committee (No. 1211–2011). Based on the cause of death, the cases were divided into three groups: Cases with noninfectious causes of death were the Control^-^ group (*n* = 11). Control^+^ cases (*n* = 5) died from severe infections. In none of the SIDS cases (*n* = 26), specific causes of death were found following thorough post-mortem and death scene investigations. Tables 1 and 2 provide detailed information on control and SIDS cases. Thymus tissue samples were collected during the autopsy and preserved at -80°C for long-term storage.

**Table 1:** Control cases with detailed information.

Coded case No.	Group	Sex	Age (weeks)	Postmortem interval (days)	Storage period (years)	Thymus weight (g)	Body weight (g)	Ratio of thymus weight to body weight (%)	Height (cm)	BMI (kg/m^2^)	Cause of death
C1-3	Control^–^	Female	1	2	9	14	2928	0.48	50	11.71	Heart defect
C2-3	Control^–^	Male	17	2	9	20	8000	0.25	66	18.37	Trauma
C6-3	Control^–^	Male	21	2	9	30	6100	0.49	65	14.44	Heart defect
C13-3	Control^–^	Female	25	2	5	50	8850	0.56	68	19.14	Metabolic disorder (LCHAD defect)
C-S16-3	Control^–^	Female	10	1	8	24	3980	0.60	52	14.72	Trauma & cardiomyopathy
C14-3	Control^–^	Male	9	2	2	28	5684	0.49	61	15.28	Trauma
C16-3	Control^–^	Male	0	1	2	6	2500	0.24	47	11.32	Death during birth (umbilical knot)
C17-3	Control^–^	Male	5	3	2	22	5300	0.42	57	16.31	Trauma
C18-3	Control^–^	Female	8	2	1	14	4648	0.30	57	14.31	Shaken impact syndrome
C20-3	Control^–^	Male	65	3	1	18	9000	0.20	71	17.85	Trauma
C21-3	Control^–^	Male	0	2	1	16	2350	0.68	47	10.64	Premature placental ablation
C8-3	Control^+^	Male	15	1	9	20	5600	0.36	56	17.86	Trauma & aspiration pneumonia
C10-3	Control^+^	Male	23	3	8	48	10,800	0.44	66	24.79	Aspiration pneumonia
C3-3	Control^+^	Female	97	2	9	34	12,000	0.28	87	15.85	Sepsis
C7-3	Control^+^	Male	8	1	9	4	2974	0.13	43	16.08	Sepsis
C12-3	Control^+^	Female	63	4	8	14	12,200	0.11	80	19.06	Sepsis

The Control^-^ represents control cases without symptoms of infection. The Control^+^ group represents control cases who died from severe infection.

**Table 2:** SIDS cases with detailed information.

Coded case No.	Group	Sex	Age (weeks)	Postmortem interval (days)	Storage period (years)	Thymus weight (g)	Body weight (g)	Ratio of thymus weight to body weight (%)	Height (cm)	BMI (kg/m^2^)	Autopsy findings	Clinical infection history	Infection duration in history	Sleeping position	Co-sleeping	Maternal smoking	Other risk factors
S2-3	SIDS^-^	Female	22	2	10	48	10,100	0.48	69	21.21	–	–	–	Prone	No	Yes	–
S3-3	SIDS^-^	Male	13	1	9	50	6900	0.72	62	17.95	–	–	–	Prone	No	Unknown	–
S7-3	SIDS^-^	Female	3	2	9	24	4100	0.59	55	13.55	–	–	–	Prone	No	Unknown	–
S9-3	SIDS^-^	Male	14	4	9	34	6400	0.53	63	16.12	–	–	–	Prone	Yes	Unknown	–
S10-3	SIDS^-^	Female	36	1	9	34	6400	0.53	63	16.12	–	–	–	Prone	No	Unknown	Preterm
S11-3	SIDS^-^	Male	7	2	9	16	5100	0.31	61	13.71	–	–	–	Back	No	Unknown	Preterm
S13-3	SIDS^-^	Female	19	2	8	16	3500	0.46	49	14.58	–	–	–	Prone	No	Unknown	Preterm (26 weeks)
S15-3	SIDS^-^	Male	5	1	8	30	4200	0.71	54	14.40	–	–	–	Back	Yes	Unknown	–
S18-3	SIDS^-^	Male	9	2	8	28	4350	0.64	54	14.92	–	–	–	Unknown	No	Unknown	–
S22-3	SIDS^-^	Male	18	2	7	44	6595	0.67	63	16.62	–	–	–	Prone	No	Unknown	–
S23-3	SIDS^-^	Male	12	2	7	30	7800	0.38	65	18.46	–	–	–	Prone	No	Unknown	–
S25-3	SIDS^-^	Female	11	1	6	34	5720	0.59	60	15.89	–	–	–	Prone	No	Unknown	–
S26-3	SIDS^-^	Male	10	2	6	36	6015	0.60	61	16.17	–	–	–	Back	No	Yes	–
S27-3	SIDS^-^	Male	30	2	6	38	8960	0.42	69	18.82	–	–	–	Prone	No	Unknown	–
S28-3	SIDS^-^	Male	23	4	6	50	8465	0.59	70	17.28	–	–	–	Back	Yes	Unknown	–
S29-3	SIDS^-^	Male	23	1	6	55	9350	0.59	71	18.55	–	–	–	Prone	No	Unknown	–
S30-3	SIDS^-^	Female	8	2	6	30	4007	0.75	53	14.26	–	–	–	Prone	No	Unknown	–
S32-3	SIDS^+^	Male	17	2	3	52	8000	0.65	65	18.93	–	–	–	Side	No	Unknown	–
S5-3	SIDS^+^	Female	28	2	9	24	7600	0.32	67	16.93	Otitis media	Otitis media	2 weeks	Prone	No	Unknown	–
S6-3	SIDS^+^	Male	11	2	9	44	5900	0.75	61	15.86	–	Fever	5 days	Prone	Yes	Unknown	–
S8-3	SIDS^+^	Female	14	2	9	28	6000	0.47	65	14.20	–	Respiratory infection	4 weeks	Back	No	Unknown	–
S12-3	SIDS^+^	Male	6	3	8	26	4600	0.57	53	16.38	Tracheobronchitis	Fever	2 days	Prone	No	Unknown	–
S17-3	SIDS^+^	Male	19	2	8	26	6100	0.43	64	14.89	–	Respiratory infection	2 weeks	Back	No	Unknown	–
S20-3	SIDS^+^	Female	13	1	8	14	4342	0.32	55	14.35	–	Rota virus	3 weeks	Back	No	Unknown	–
S21-3	SIDS^+^	Male	16	3	7	36	5400	0.67	60	15.00	Tracheobronchitis	Respiratory infection	1 week	Prone	Yes	Unknown	–
S24-3	SIDS^+^	Male	29	1	6	36	7720	0.47	72	14.89	Tonsillitis	–	–	Prone	No	Unknown	–

The SIDS^–^ group represents SIDS cases with no symptoms of infection. The SIDS^+^ group represents SIDS cases with observed symptoms of infection or a documented history of infection.

### Measurement of cytokines using Multiplex arrays

The Bio-Rad cell lysis Kit (#171304011, BioRad, Hercules, CA) was used to extract protein from thymus tissue specimens. To determine the protein concentration, the PierceTM BCA Protein Assay Kit (#23225, Thermo Scientific, Waltham, MA) was used. The concentration was adjusted to 1000 μg/mL with Sample Diluent (BioRad, Hercules, CA).

The concentrations of cytokines were measured in thymus protein lysates containing 50 µg protein using the Bio-Plex ProTM Human Cytokine 27-Plex Assay (#500KCAF0Y, BioRad, Hercules, CA) following the manufacturer’s instructions. “*Key biomarkers of inflammation from the TNF superfamily proteins, IFN family proteins, Treg cytokines, and MMPs can be measured using a single multiplex kit*.” (https://www.bio-rad.com). These 27 cytokines are classified into four groups according to their biological functions in the inflammatory response (Table 3).

**Table 3:** 27 cytokines included in the study.

Cytokine groups	Specific cytokines in each group								
Pro-inflammatory cytokines	IL-1β	IL-2	IL-6	IL-7	IL-12 (p70)	IL-15	IL-17	IFN-γ	TNF-α
Pro-inflammatory chemokines	CCL1 (MCP-1)	CCL3 (MIP-1α)	CCL4 (MIP-1β)	CCL5 (RANTES)	CCL11 (Eotaxin)	CXCL10 (IP-10)	CXCL8 (IL-8)		
Anti-inflammatory mediators	IL-1RA	IL-4	IL-5	IL-9	IL-10	IL-13			
Growth factors	FGF basic	G-CSF	GM-CSF	PDGF-bb	VEGF				

The Bio-Plex-Manager 6.2 software (BioRad, Hercules, CA) was used to calculate the standard curves and concentrations. If the concentration was below the lower limit of detection (LLOD), it was assigned the value of LLOD, and if it was beyond the upper limit of detection (ULOD), it was assigned the value of ULOD.

### Statistical analysis

The distribution of variables was analyzed with the Shapiro-Wilk (S-W) normality test. A Mann–Whitney U test was used to compare two groups of continuous variables. A Chi-square (*x*^2^) test was used to determine the statistical significance of the proportion of males and females in two groups. The age-related correlation was examined using a Spearman’s rank correlation analysis.

To obtain a general understanding of the age-related alterations in the cytokine/chemokine milieu in the human thymus of infants less than 52 weeks, mean-centered scaled concentrations of cytokines, as proposed in our previous study^37^, were used. To achieve this, we calculated the mean value for each cytokine across all samples and expressed individual results as a percentage of the mean. The sum of all 27 cytokines was then determined for each sample.

To reduce the influence of age, a confounding variable, an age-layered analysis was performed to calculate the differences in cytokines levels between the two groups. The cutoff age was established based on the intersection of the age-related correlation lines between Control^-^ and SIDS groups (Thymus: 20 weeks based on Fig. 3).

A *p* value < 0.05 (two-tailed) indicates statistical significance. All statistical analyses were conducted by SPSS 24.0 (SPSS Inc. Chicago) or Graphpad Prism 9.0 (Graphpad Software, San Diego).

### Histological and immunohistochemistry (IHC) studies

A subset of cases was analyzed histopathologically. Thymic tissue of five SIDS and five control cases (age-matched) was formalin-fixed and paraffin-embedded. Analysis of the distribution of immune cells was performed by light microscopy. Therefore, hematoxylin-eosin (HE) staining and IHC of all 10 cases was done, including CD4 and CD8 (both Roche, Basel, Switzerland) for T lymphocytes, CD 138 (Dako, Glostrup, Denmark) for plasma cells and CD68-PGM-1 (Dako, Glostrup, Denmark) for macrophages.

## Results

### Baseline analysis

To control the confounding factors, an initial baseline analysis was carried out (Table 4). There were significant differences in thymus weight (*p* = 0.014) and the ratios of thymus weight to body weight (*p* = 0.004) between the Control^-^, Control^+^, and SIDS groups, showing higher thymic weight in SIDS. There were no significant differences between the three groups in age, sex proportion, body weight, body height, or body mass index (BMI).

**Table 4:** Baseline analysis of participants.

Characteristics	Control^-^ (*n* = 11)	Control^+^ (*n* = 5)	SIDS (*n* = 26)	*p* value
Age in weeks, median (range)	9 (0–65)	23 (8–97)	14 (3–36)	n.s.
Male, %	64%	60%	65%	n.s.
Thymus weight in grams, median(range)	20 (6–50)	20 (4–48)	34 (14–55)	0.014
Body weight in grams, median (range)	5300 (2350–9000)	10,800 (2974–12,200)	6058 (3500–10,100)	n.s.
Body height in cm, median (range)	57 (47–68)	66 (43–87)	62.5 (49–72)	n.s.
Ratios of thymus weight to body weight (%), median (range)	0.48 (0.20–0.68)	0.28 (0.11–0.44)	0.58 (0.31–0.75)	0.004
Body Mass Index in kg/m^2^, median (range)	14.72 (10.64–19.14)	17.86 (15.85–24.79)	16 (13.55–21.21)	n.s.

n.s. represents no significance in statistics. The Control^-^ represents control cases without symptoms of infection. The Control^+^ group represents control cases who died from severe infection.

### Thymic cytokine profiling in controls with severe infection (Control^+^)

In the next step we compared the cytokine levels in our 3 groups (Table 5). There were 14 elevated cytokines (IL-1 β, -2, -4, -5, -6, -10, CXCL8, CXCL10, CCL1, CCL3, CCL11, FGF basic, G-CSF, and TNF-α) and one decreased ratio of IL-1RA to IL-1β in the Control^+^ group, compared to the Control^-^ group (Table 5 left).

**Table 5:** Statistical differences in the cytokine level of SIDS cases and controls.

Cytokines	Cytokine concentration (pg/mL)									
	Control^-^ (*n* = 11)		Control^+^ (*n* = 5)		SIDS (*n* = 26)		Trends for Control^+^, compared to Control^-^	*p* value	Trends for SIDS, compared to Control^-^	*p* value
	Median	Range (minimum-maximum)	Median	Range (minimum-maximum)	Median	Range (minimum-maximum)				
IL-1RA/IL-1β	1419.43	237.75–3288.34	162.89	44.35–266.12	363.78	184.91–685.21	**↓**	**0.002**	**↓**	**0.008**
IL-1β	3.37	0.6–28.69	109.14	24.04–420.98	20.10	10.97–40.86	↑	**0.002**	↑	**0.036**
IL-1RA	4778.72	1305.88–14194.7	11050.49	5184.56–18672.52	7595.99	3576.21–19555.97		0.069		0.195
IL-2	4.47	1.19–29.12	34.31	18.66–135.01	22.98	16.8–31.56	↑	**0.027**	↑	**0.017**
IL-4	0.83	0.29–7.18	6.42	5.13–11.09	5.80	4.74–7.76	↑	**0.038**	↑	**0.023**
IL-5	30.22	6.38–423.64	329.94	214–524.62	231.54	164.48–355.55	↑	**0.013**	↑	**0.032**
IL-6	15.09	1.31–160.33	13701.05	137.69–21161.43	64.04	33.17–367.32	↑	**0.001**		0.058
IL-7	15.71	7.38–24.32	20.05	8.38–32.71	15.91	5.84–75		0.913		0.752
CXCL8 (IL-8)	45.82	6.73–330.57	2451.54	76.07–37479.32	76.77	41.61–594.02	↑	**0.005**		0.144
IL-9	31.59	4.17–143.75	113.58	81.34–175.93	125.72	93.21–205.92		0.221	↑	**0.003**
IL-10	1.24	0.1–4.84	4.17	3.18–8.82	3.35	2.37–7.42	↑	**0.013**	↑	**0.005**
IL-12 (p70)	2.44	0.67–17.21	8.54	3.78–16.39	10.33	3.34–22.05		0.221	↑	**0.035**
IL-13	9.93	1.66–38.71	10.17	3.9–53.01	17.68	7.01–54		0.661	↑	**0.020**
IL-15	42.74	11.37–190.36	134.96	121.8–233.72	144.60	110.96–216.9		0.377		0.084
IL-17	9.07	2.57–35.04	33.17	25.74–49.58	27.60	20.2–37.38		0.052		0.060
CCL11 (Eotaxin)	2.02	0.53–27.7	28.52	11.98–48.48	22.11	12.71–63.13	↑	**0.027**	↑	**0.002**
FGF basic	188.02	35.89–807.75	650.49	573.89–1257.22	712.25	286.29–1661.31	↑	**0.013**	↑	**0.001**
G-CSF	669.29	327.8–946.69	17793.88	838.68–46018.13	810.58	627.61–1111.71	↑	**0.002**	↑	**0.014**
GM-CSF	2.84	0.89–14.9	11.43	8.63–75.26	11.24	7.03–16.21		0.115	↑	**0.033**
IFN-γ	5.78	2.4–106.81	157.93	59.91–217.26	63.33	37.34–94.88	↑	**0.003**	↑	**0.032**
CXCL10 (IP-10)	1113.76	151.8–4482.72	6321.40	1532.46–87765.67	2733.60	1415.57–6949.32	↑	**0.009**		0.073
CCL1 (MCP-1)	76.80	10.8–987.27	6178.34	237.75–24282	107.60	60.62–833.59	↑	**0.001**		0.084
CCL3 (MIP-1α)	592.95	131.09–3149.37	577.89	296.33–2739.39	682.13	210.98–2904.2		0.827		0.370
PDGF-bb	3.01	1.54–60.97	49.18	25.79–85.77	47.58	27.91–97.68		0.052	↑	**0.007**
CCL4 (MIP-1β)	122.54	32.46–494.29	390.10	157.56–837.52	279.54	197.46–674.96		0.09	↑	**0.018**
CCL5 (RANTES)	266.10	29.08–2195.66	662.94	508.49–1107.37	1549.49	604.65–6920.89		0.583	↑	**0.002**
TNF-α	14.31	3.4–123.82	108.59	44.89–326.7	67.00	49.33–169.33	↑	**0.038**	↑	**0.025**
VEGF	99.00	35.37–262.66	212.64	175.56–274.23	210.84	165.86–348.08		0.267		0.097

*p* value < 0.05 marked in bold. The Mann–Whitney U test was used to compare two groups of continuous variables. The Control^-^ represents control cases without symptoms of infection. The Control^+^ group represents control cases who died from severe infection.

#### Thymic cytokines and weight in SIDS

Comparison between the Control^-^ and SIDS groups, on the other hand, revealed statistically significant changes in 17 out of 27 cytokines (IL-1β, -2, -4, -5, -9, -10, -12, -13, CCL4, -5, -11, FGF basic, G-CSF, GM-CSF, IFN-, PDGF-bb, and TNF-α) and the ratio of IL-1RA to IL-1 (Table 5 right). Thus, most cytokines increased in the septic Control^+^ group were also increased in SIDS. However, at least for most proinflammatory cytokines such as IL-6, IL-8 or TNF- α, the magnitude of these changes in SIDS was lower than that in the Control^+^ group.

**Fig. 1:**
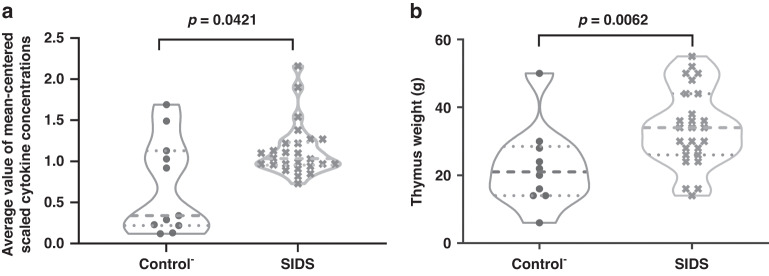
Comparison of mean-scaled cytokine concentrations and thymic weight between the groups of Control^-^ and SIDS in violin plots. **a** Comparison of mean-scaled cytokine levels. **b** Comparison of thymic weight. The dash line in each group is the value of 25, 50 (in bold), or 75 percentiles. A *p* value < 0.05 indicates statistical significance. The Control^-^ represents control cases without symptoms of infection.

Moreover, normalized averages of all cytokines (*p* = 0.0421) were higher in the thymus of SIDS compared to those of Control^-^ (Fig. 1a), indicating an activated immune system. Similarly (Fig. 1b), greater thymus weight was seen in SIDS cases (*p* = 0.0062) compared to Control^-^.

For further analysis we concentrated only on the comparison of the SIDS and Control^-^ groups while we did not display results for the septic Control^+^ group.

#### Impaired age-dependent thymic cytokines and weight in SIDS

**Fig. 2:**
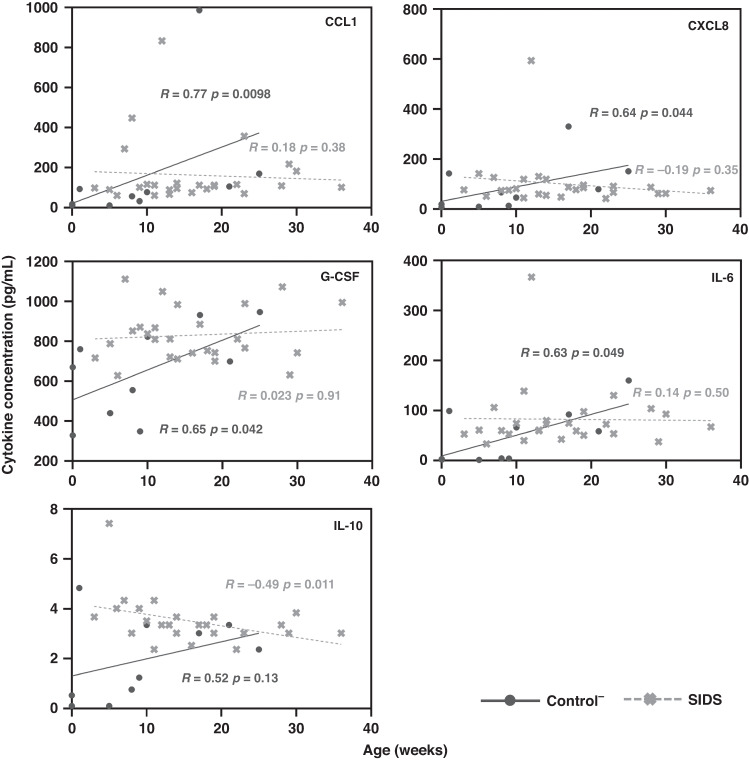
Correlation analyses between cytokine concentrations and age in the groups of Control^-^ and SIDS. Correlations of 5 cytokines (CCL1, CXCL8, G-CSF, IL-6, and IL-10) with age were compared in Control^-^ and SIDS cases. A *p* value < 0.05 indicates statistical significance. The Control^-^ represents control cases without symptoms of infection.

**Fig. 3:**
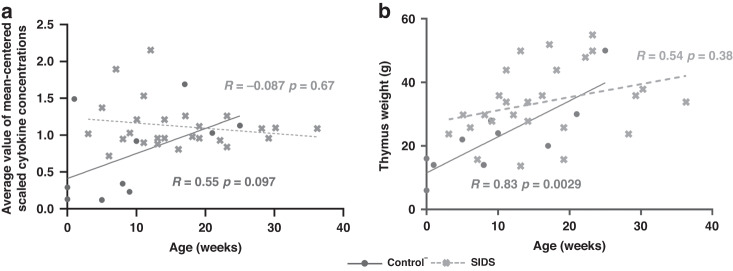
Age-dependent analyses on the mean-scaled cytokine concentrations and thymic weight between the groups of Control^-^ and SIDS. **a** Age-dependent analyses on the mean-scaled cytokine levels. **b** Age-dependent analysis on the thymic weight. A *p* value < 0.05 indicates statistical significance. The Control^-^ represents control cases without symptoms of infection.

When cytokine concentrations were plotted against age (Fig. 2), four cytokines were positively correlated with age in Control^-^ but not SIDS cases (CCL1, CXCL8, G-CSF, and IL-6). IL-10 was negatively correlated with age in SIDS, but not Control^-^. When the normalized means for all cytokines were plotted against age (Fig. 3a), a borderline (positive) correlation was found in the Control- group (R = 0.55, *p* = 0.097), but not in SIDS cases (R = –0.087, *p* = 0.67). In the Control^-^ group, there was a correlation between thymus weight and age (R = 0.83, *p* = 0.0029), but not in SIDS (R = 0.54, *p* = 0.38) (Fig. 3b). However, no statistical association was discovered between the cytokine concentrations and thymus weight (data not shown). To further evaluate the age differences, the Control^-^ and SIDS cases were respectively split into two age groups. As shown in Table 6, elevated cytokine levels in SIDS, compared to Control^-^, were mainly found in the 0–20 weeks age group but not the group older than 20 weeks.

**Table 6:** Statistical differences in the thymic weight and cytokine level of the age subgroups of SIDS cases and controls.

Cytokines & Thymic weight	0 <Age≤20 weeks						Age >20 weeks					
	Control^-^ (*n* = 8)		SIDS (*n* = 19)		Trends	*p* value	Control^- ^(*n* = 3)		SIDS (*n* = 7)		Trends	*p* value
	Cytokine levels in pg/mL& Thymic weight in gram						Cytokine levels in pg/mL& Thymic weight in gram					
	Median	Range (Min.-Max.)	Median	Range (Min.-Max.)			Median	Range (Min.-Max.)	Median	Range (Min.-Max.)		
Thymic weight	18	6–28	30	14–52	↑	**0.001**	30	18–50	38	24–55		0.383
IL-1RA/IL-1β	1904.52	288.67–3288.34	349.66	184.91–685.21	**↓**	**0.004**	499.17	237.75–2864.32	370.68	213.42–619.91		0.667
IL-1β	2.40	0.6–28.69	21.62	10.97–40.86	↑	**0.034**	20.10	0.76–20.41	17.20	13.02–27.8		1.000
IL-1RA	4672.49	1305.88–14194.7	7340.16	3576.21–19555.97		0.307	4778.72	2176.88–10188.13	7851.82	4646.73–10049.38		0.667
IL-2	3.83	1.19–29.12	22.98	16.8–31.56	↑	**0.016**	20.82	2.22–23.59	22.98	16.8–24.52		0.667
IL-4	0.75	0.29–7.18	5.77	4.74–7.76		0.051	5.47	0.39–5.65	5.83	4.98–6.3		0.117
IL-5	27.45	6.38–423.64	232.88	164.48–355.55	↑	**0.034**	208.58	21.26–259.52	224.81	192.19–246.24		0.833
IL-6	3.94	1.31–99.22	60.70	33.17–367.32		0.075	58.53	15.09–160.33	72.56	37.58–130.2		0.833
IL-7	18.47	7.38–24.32	16.51	5.84–75		0.979	12.44	9.22–24.32	15.71	9.22–34.22		0.517
CXCL8 (IL-8)	32.41	6.73–330.57	80.89	44.26–594.02		0.075	78.71	26.58–150.93	66.29	41.61–90.73		0.833
IL-9	26.04	4.17–143.75	126.70	94.52–205.92	↑	**0.011**	94.20	20.95–125.06	120.14	93.21–132.93		0.267
IL-10	1.00	0.1–4.84	3.35	2.37–7.42	↑	**0.011**	2.37	0.53–3.35	3.02	2.37–3.84		0.383
IL-12 (p70)	1.97	0.67–13.12	10.01	3.34–22.05	↑	**0.039**	6.84	1.38–17.21	12.29	5.98–13.94		0.667
IL-13	6.99	1.66–38.71	17.44	7.01–54	↑	**0.013**	18.91	2.26–18.91	18.17	13.99–29.74		1.000
IL-15	38.65	11.37–190.36	145.23	110.96–216.9		0.106	137.55	18.16–150.29	134.96	113.7–188		0.667
IL-17	7.20	2.57–35.04	27.60	20.2–37.38		0.066	27.13	2.86–28.76	27.60	24.82–31.31		0.517
CCL11 (Eotaxin)	1.83	0.53–27.7	22.18	12.71–63.13	↑	**0.001**	24.63	0.8–27.22	21.83	14.06–34.5		0.667
FGF basic	164.89	35.89–807.75	723.19	465.47–1661.31	↑	**0.004**	477.33	80.34–502.06	607.05	286.29–1069.71		0.183
G-CSF	612.52	327.8–931.95	809.67	627.61–1111.71	↑	**0.034**	698.40	570.47–946.69	811.48	631.13–1073.17		0.267
GM-CSF	2.70	0.89–14.9	10.33	7.03–16.21		0.066	9.85	1.24–12.05	11.66	9.37–14.16		0.267
IFN-γ	5.08	2.4–106.81	63.33	37.34–94.88	↑	**0.045**	56.49	5.78–68.24	63.33	44.82–75.86		0.383
CXCL10 (IP-10)	1056.55	151.8–4482.72	2823.14	1415.57–6949.32	↑	**0.029**	3119.01	304.49–3288.69	2707.22	1495.01–3490.88		1.000
CCL1 (MCP-1)	44.17	10.8–987.27	101.04	60.62–833.59	↑	**0.011**	169.91	105.77–216.67	115.61	69.54–357.08		1.000
CCL3 (MIP-1α)	532.81	131.09–3149.37	595.80	210.98–2904.2		0.621	592.95	132.58–1654.48	831.79	390.59–1960.21		0.517
PDGF-bb	2.38	1.54–60.97	45.98	30.02–97.68	↑	**0.016**	40.64	1.54–44.91	50.25	27.91–94.43		0.267
CCL4 (MIP-1β)	92.79	32.46–494.29	278.56	197.46–674.96	↑	**0.019**	259.95	56.83–287.73	286.04	212.07–314.18		0.667
CCL5 (RANTES)	199.23	29.08–2195.66	1556.15	604.65–6920.89	↑	**0.003**	1236.94	142.2–1314.73	1485.30	829.63–2142.64		0.183
TNF-α	12.33	3.4–123.82	69.57	49.33–169.33	↑	**0.034**	55.98	6.34–78.36	64.44	58.92–72.87		0.517
VEGF	94.18	35.37–262.66	216.21	165.86–348.08		0.095	199.94	39.46–243.26	189.77	166.83–264.33		1.000

*p* value < 0.05 marked in bold. The Mann–Whitney U test was used to compare two groups of continuous variables. The Control^-^ represents control cases without symptoms of infection.

### Immunohistochemistry (IHC) examination on the thymus of SIDS

SIDS and Control^-^ cases consisted of morphologically normal thymic tissue and had the same light microscopic immunoprofile (supplemental Figure S1). Nevertheless, as the slides of the originally deep-frozen tissue was of low quality, these examinations were confined to 10 samples and the conclusions drawn from these are limited.

## Discussion

The thymus plays an essential role in the immune development of infants.^18^ As the immune system is considered to be involved in the etiology of SIDS, we hypothesized that the morphology and function of the thymus of SIDS infants differ from other deaths. However, earlier morphological SIDS studies reported controversial findings on the thymus.^23–28,31,32^ We thus performed for the very first time a study on cytokines and other inflammatory mediators in the thymic tissue. We discovered elevated thymic cytokine concentrations and weights in SIDS cases, but as opposed to the controls, the SIDS cases showed no increase of the cytokines with age: Thus, increased concentrations were only detectable before the 20^th^ week of life (and thus in the peak prevalence of SIDS) (Table 6).

The Control^+^ group who comprised deaths from severe infection had elevated levels of 14 cytokines compared to the Control^-^ group who died without septic diseases (Table 5), including critical pro-inflammatory mediators such as IL-1β, IFN-γ, and TNF-α, which were largely in line with increased cytokine in the cerebrospinal fluid (CSF) of infants that died of viral infections^38^. Also, in the baseline analysis, the ratio of thymus weight ratio to body weight was decreased in the controls with severe infections (Table 4). These findings, that we considered to be the consequence of glucocorticoids and up-regulated inflammatory mediators,^39–41^ are in line with previous studies regarding infection-induced thymic atrophy or involution.^23,42–45^ In the context of our study, we not only used the Control^+^ group to confirm the reliability of the adopted cytokine measurement method, but also to assess the question whether SIDS – without pathologic anatomical findings of an infection – shows an activation of the immune system similar to that in septic deaths.

When comparing the differences between the SIDS and Control^-^ groups, 17 cytokines, and the thymus weight were increased in SIDS (Table 5). Like for the Control^+^ group, important pro-inflammatory mediators associated with the infection, including IL-1β, IFN-γ, and TNF-α, were highly expressed. In addition, when compared to Control^-^, ten significant increased cytokines overlapped between the SIDS and Control^+^ groups. Altogether, the overlapped cytokine levels were highest in the Control^+^ group, similar in distribution but mostly of lower magnitude in SIDS infants, and lowest in the Control^-^ group (Table 5). Also, most of these up-regulated cytokines are considered to boost the thymic development, and hence T cell development.^34,35^ For us, this observation implies that there may be a mild to moderate degree of infection causing increased intrathymic cytokines in SIDS, which in turn induces reactive thymic hyperplasia, as already suggested, e.g. by Goldwater et al.^27^ The increased thymus weight found in our study (and some, albeit not all, others^25,27,32^) might be explained by this mechanism. As we know, SIDS cases with a case history of slight infection or postmortem detected upper airway infections are only a fraction of the overall caseload. However, we unexpectedly found no statistically significant differences in thymic cytokine concentrations and thymus weights between SIDS with and without weak infections (data not shown). This just provides the possibility that subtle, easily overlooked, or undetected subclinical infections may be widely prevalent in the SIDS group, causing no obvious differences of cytokines between SIDS cases with or without “observed” infection.

Increasing evidence suggests that transitional, age-dependent disorders (causing a “critical developmental stage” according to the triple risk theory) may be associated with the pathogenesis of SIDS. Our previous publication on the lungs found an impaired age-dependent cytokine network in SIDS, leading to an underlying disturbed immune response state.^37^ The increasing levels of cytokines during the ageing of healthy infants are considered to be the consequence of a “trained” immune system, due to increasing contacts with microorganisms after birth.^46,47^ Thus, the normal development of the thymus is accompanied by age-related alterations in cytokine expression and thymic weights.^48,49^

In this study, thymic weight, and cytokines (e.g., CCL1, CCL8, G-CSF and IL-6) increased with age in infants that died without serious infectious diseases (Control^-^). In SIDS, on the other hand, we found higher thymic weights and cytokines in infants up to 20 weeks old, but no age-dependent changes. To us, this suggests that infection or other immunologic challenges in SIDS cause elevated cytokine levels during earlier infancy, but also might repress the proper development of the immune system. The higher thymic weight and cytokine levels might thus reflect subclinical infections, mainly present in the first 5 months of life in SIDS (Table 6). Intriguingly, a Canadian study based on data from 900 infant autopsies also demonstrated that the thymus weight of SIDS/ sudden unexpected death syndrome (SUDS) in the infants younger than 25 weeks cases is greater than the counterpart controls’ one.^25^ Even more so, this age group includes the peak prevalence (2 ~ 4 months) of SIDS. The reasons why SIDS infants are, at a vulnerable period, more prone to succumb to common infections than other infants are still not well known, although several studies tried to interpret a dysfunctional immune status from a genetic viewpoint.^6,50–53^

Since increased cytokines observed in the thymus of SIDS might be reflected by morphologic changes, such as reactive hyperplasia, age-matched histological examination on composition of thymic immune cells was performed but no significant differences were found between SIDS and Control^-^ groups. Nevertheless, we could not rule out the presence of an altered thymic output in SIDS although this has not been formally shown so far. Thus, it would be interesting to explore whether the altered cytokine and chemokine profile in SIDS thymus tissue would also impair thymic T cell development and the T cell repertoire (TCR) both for CD4 and CD8 T cells. Some further experimental approaches, such as single cell sequencing, quantification of T cell receptor repertoire and TCR excision circles (TRECs), might be needed to investigate cell composition and thymic function, which would assist in understanding the potential role of the thymic output in SIDS.

Several of limitations of this study should be mentioned herein. Firstly, a limited number of cases was recruited for this study, which causes reduced statistical test efficacy especially in the subgroup analysis on risk factors like birth weight, preterm birth, family history, maternal risk, etc. The analysis on the association between risk factors and cytokine levels showed no significant results (data not shown). Secondly, a study on postmortem material has to rely on samples from other deceased persons. These are not ideal as controls (healthy infants would be) and the interpretation of our results might be biased by that fact. Finally, it should be kept in mind that postmortem cytokine levels may be affected by postmortem changes (e.g., autolysis and putrefaction) because the postmortem interval between the onset of death and tissue sampling cannot be avoided. Therefore, the possible effects of postmortem changes should be considered when interpreting the relevant data.

## Conclusion

In summary, elevated cytokines, as well as thymus weight, and impaired thymic age-dependent changes in SIDS may be influenced by subclinical infections. The increase in thymic cytokine levels and thymic weight were mainly present in SIDS cases under 5 months of age, which overlaps with the high incidence of SIDS onset (2 ~ 4 months), suggesting that subclinical infection might play an important role in the onset of SIDS among young susceptible infants.

### Supplementary information

Supplementary Information

## Data Availability

The datasets generated during and/or analyzed during the current study are available from the corresponding author on reasonable request.
