# Supplementary material for: Age-related cytokine imbalance in the thymus in sudden infant death syndrome (SIDS)
Source: Pediatr Res. 2023 Sep 7;95(4):949–58. doi: 10.1038/s41390-023-02809-6 (PMC10920197; doi:10.1038/s41390-023-02809-6)
Supplement: Supplementary file 1 — Supplementary Information [file 41390_2023_2809_MOESM1_ESM.pdf]

Age-related cytokine imbalance in the thymus in sudden infant death syndrome (SIDS)

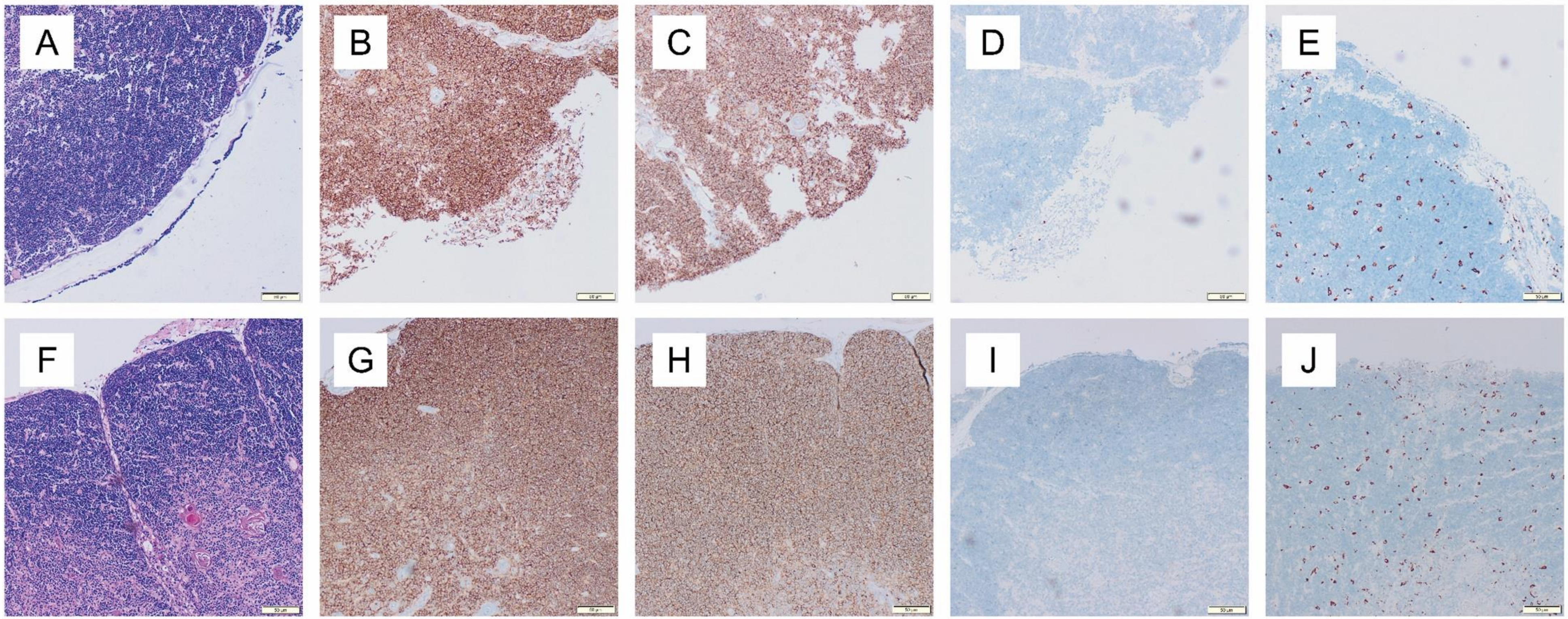

Supplemental Figure S1 Histological staining of thymic tissue of a SIDS case (A-E) and a control case (F-J). A, F: HE-staining; B, G: CD4-staining; C, H: CD8-staining; D, I: CD138-staining; E, J: PGM-1-staining.
